# *INI1*-Intact Sinonasal Carcinoma with Rhabdoid Features

**DOI:** 10.1155/2021/6075130

**Published:** 2021-11-18

**Authors:** Erin Mulry, Danielle M. Blake, Poornima Hegde, Todd E. Falcone

**Affiliations:** ^1^Department of Surgery, Division of Otolaryngology—Head and Neck Surgery, University of Connecticut, Farmington, CT, USA; ^2^Department of Otolaryngology, RWJ Barnabas Health-Cooperman Barnabas Medical Center, Livingston, NJ, USA; ^3^Department of Anatomical Pathology, University of Connecticut, Farmington, CT, USA

## Abstract

Sinonasal malignancies are known for their associated poor prognosis and diversity of histologic features. While poor prognosis is largely due to advanced disease at presentation, histologic features also play a significant role. Therefore, accurate pathologic diagnosis is of utmost importance. Here, we describe a 63-year-old male with chronic left-sided nasal obstruction and left-sided epistaxis who was found to have a large mass occupying most of the nasal cavity extending through the nasopharynx to just below the nasopharyngeal surface of the soft palate. During surgical excision, the mass was noted to originate from the floor of the maxillary sinus with erosion of the medial wall of the maxillary sinus. Pathology revealed a diagnosis of INI1-intact poorly differentiated composite carcinoma with rhabdoid phenotype and sarcomatoid and squamous cell carcinoma foci arising within an inverted papilloma. Included in this report is a detailed description of both the patient's medical course and this pathologically novel sinonasal neoplasm. We aim to elucidate this rare tumor's complex features in order to improve future diagnosis and stimulate prospective research on sinonasal malignancies with complex histology.

## 1. Introduction

While accounting for less than 0.5% of all malignancies, sinonasal malignancy remains an important disease, as it tends to be associated with a poor prognosis [1]. While this is largely attributed to advanced disease at diagnosis, prognosis is also affected by histologic subtype, grade, and local invasion [1]. A majority of sinonasal carcinomas are squamous cell carcinomas (SCC), which can develop de novo or through malignant transformation of inverted Schneiderian papillomas [2].

Here, we present what appears to be the first case of a patient with poorly differentiated composite sinonasal carcinoma with rhabdoid phenotype, focal sarcomatoid, and SCC arising within an inverted papilloma.

## 2. Case Presentation

**Figure 1:**
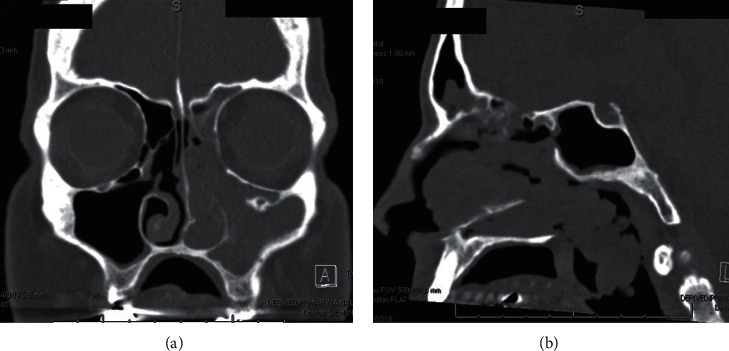
Coronal CT of sinonasal mass with complete opacification of left paranasal sinuses (a) and sagittal CT of mass extending from the nasal cavity down to oropharynx in contact with the soft palate (b).

A 63-year-old male nonsmoker with a history of type 2 diabetes, hyperlipidemia, and hypertension presented with left-sided nasal obstruction and intermittent left-sided epistaxis for a duration of several years. He denied history of trauma or dental work, as well as tooth pain, facial numbness, or past sinus surgery. Physical exam was notable for a mass in the oropharynx just inferior to the soft palate extending down from the nasopharynx. Nasal endoscopy revealed a large fleshy and friable mass in the left nasal cavity obstructing the view of the middle turbinate and touching the nasal floor, causing complete nasal obstruction. Computed tomography (CT) of the sinuses was concerning for a mass extending from the left maxillary sinus antrum to the nasopharynx and oropharynx with complete opacification of the adjacent paranasal sinuses (Figure 1).

The patient was taken to the operating room for left-sided endoscopic sinus surgery with complete excision of the mass for both diagnosis and treatment. The mass was firm, extremely vascular, and appeared to erode through the medial wall of the maxillary sinus from an attachment site on the floor of the maxillary sinus.

**Figure 2:**
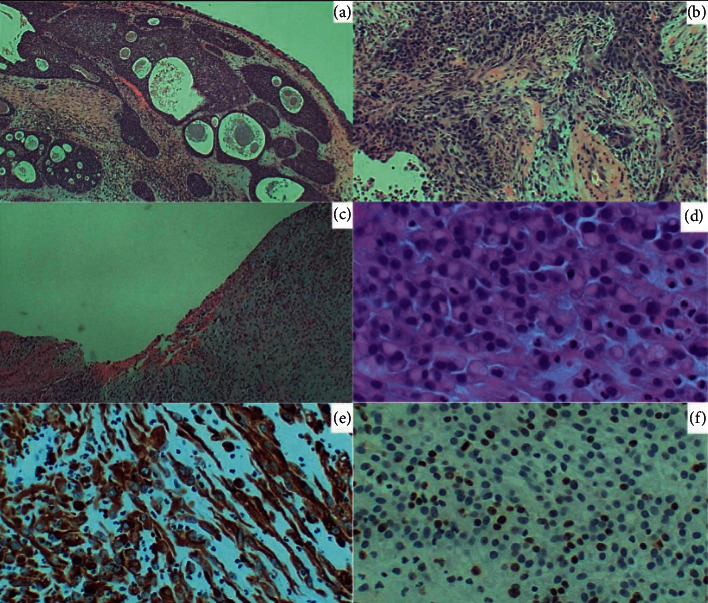
Photomicrographs of tumor sections. Sections shown above include (a) inverted papilloma (H&E, 10x); (b) poorly differentiated squamous cell carcinoma (H&E, 10x); (c) surface ulceration with underlying rhabdoid tumor phenotype (H&E, 10x); (d) rhabdoid tumor cells with prominent perinuclear acidophilic zone (H&E, 40x); (e) sarcomatoid focus within tumor and cytoplasmic globoid bodies in rhabdoid tumor cells (CAM 5.2 positive immunostain, 20x); (f) proliferative index of 20% (MIB1 immunostain, 20x).

Pathologic examination revealed a poorly differentiated composite sinonasal carcinoma with rhabdoid phenotype, foci of sarcomatoid cells, and SCC arising within an inverted papilloma (Figure 2). The tumor cells were negative for P16INK4a, a surrogate marker for HPV, and were found to retain INI-1 expression, which is typically deficient in rhabdoid tumors [3, 4].

## 3. Discussion

Given the histopathologic and immunohistochemical findings described here (Figure 2), this tumor appears to be the first reported case in the English literature of an INI1-intact sinonasal carcinoma with rhabdoid features.

SMARCB1, which codes for INI1, is a tumor suppressor gene located on chromosome 22q11.2 [3]. INI1 serves as a subunit of the switch/sucrose nonfermentable (SWI/SNF) complex, which participates in control of gene expression by remodeling chromatin [4]. Loss of INI1 expression is known to be associated with rhabdoid-type neoplasms, including the predominantly pediatric malignant rhabdoid tumors and atypical teratoid/rhabdoid tumors [3], as well as sinonasal undifferentiated carcinomas [4]. In 2014, 3 cases of sinonasal basaloid carcinoma, a subtype of poorly differentiated sinonasal carcinomas, were also reported to be INI1-deficient though only contained occasional rhabdoid cells [5].

Though few, there have been reports of rhabdoid-type tumors and sinonasal carcinomas of other types identified to be INI1-intact [3, 4]. One study reported retained INI1 expression in composite rhabdoid tumors similar to our case; however, none of the tumors in the 40 reported cases were of sinonasal origin [3]. Another study reports an INI1-intact sinonasal carcinoma that is alternatively deficient in SMARCA4, which codes for BRG1, a protein that interacts with INI1 in the SWI/SNF complex [4]. However, this INI1-intact sinonasal carcinoma was a poorly differentiated neuroendocrine carcinoma rather than rhabdoid phenotype as we report [4]. Currently, there are no reports of poorly differentiated sinonasal rhabdoid tumors with retained INI1 expression or of sinonasal rhabdoid tumors with foci of both sarcomatoid and SCC morphology.

Lastly, it is important to consider how the composite histology of this tumor impacts the prognosis. This specific tumor is undescribed in the literature, and thus predicting outcomes poses a significant challenge. Perry et al. speculated that rhabdoid cells represent a late-stage neoplasm, which is consistent with the observed poor prognosis [3]. Agaimy et al. described the death of 4 of 6 patients with SMARCA4-deficient sinonasal malignancies despite many receiving aggressive treatment [6]. Based on these observations, perhaps, we can predict that the composite histology of this tumor suggests a poor prognosis.

## 4. Conclusion

While various subtypes of sinonasal tumors are most often identified based on histologic features, poorly differentiated tumors of the sinonasal tract are known to be more difficult to classify, owing to the inherent heterogeneity of their histologic features [5]. The tumors described above, as well as the tumor we describe in our patient's case, serve to highlight the diversity of histologic findings in neoplasms of the sinonasal tract. With identification of this unique case of INI1-intact sinonasal carcinoma with rhabdoid phenotype, we aim to create awareness of its diverse features with the goal of recognizing additional cases in order to fully characterize this novel sinonasal malignancy.

## Data Availability

No data were used to support this study.
